# Signal Transduction Pathways in Breast Cancer: The Important Role of PI3K/Akt/mTOR

**DOI:** 10.1155/2020/9258396

**Published:** 2020-03-09

**Authors:** Miguel A. Ortega, Oscar Fraile-Martínez, Ángel Asúnsolo, Julia Buján, Natalio García-Honduvilla, Santiago Coca

**Affiliations:** ^1^Department of Medicine and Medical Specialities, Faculty of Medicine and Health Sciences and Networking Biomedical Research Centre on Bioengineering, Biomaterials and Nanomedicine (CIBER-BBN), University of Alcalá, Alcalá de Henares, Madrid, Spain; ^2^Ramón y Cajal Institute of Healthcare Research (IRYCIS), Madrid, Spain; ^3^Cancer Registry and Pathology Department, Hospital Universitario Principe de Asturias, Alcalá de Henares, Spain; ^4^Department of Surgery, Medical and Social Sciences, Faculty of Medicine and Health Sciences, University of Alcalá, Alcalá de Henares, Madrid, Spain

## Abstract

Breast cancer is the cancer with the highest prevalence in women and is the number-one cause of cancer mortality worldwide. Cell transduction is a fundamental process in the development and progression of cancer. Modifications in various cell signalling pathways promote tumour cell proliferation, progression, and survival. The PI3K/Akt/mTOR pathway is an example of that, and it is involved in growth, proliferation, survival, motility, metabolism, and immune response regulation. Activation of this pathway is one of the main causes of cancer cell resistance to antitumour therapies. This makes PI3K/Akt/mTOR signalling a crucial object of study for understanding the development and progression of this disease. Thus, this pathway may have a role as a potential therapeutic target, as well as prognostic and diagnostic value, in patients with breast cancer. Despite the existence of selective PI3K/Akt/mTOR pathway inhibitors and current clinical trials, the cellular mechanisms are not yet known. The present review aims to understand the current state of this important disease and the paths that must be forged.

## 1. Introduction: Current State of the Disease

Breast cancer is the most prevalent cancer type in women as well as the leading cause of cancer mortality in this population worldwide, with a peak incidence between 45 and 65 years of age [1]. Although it is not common, breast cancer can also occur in men, with a frequency of 1 in 100 diagnosed cases, representing less than 1% of all cancers in men [2].

Among the most important risk factors associated with breast cancer are ageing, family history, nulliparity, hormonal factors, such as early menarche or late menopause, and other factors related to lifestyle, such as alcohol consumption, obesity, and physical inactivity [3, 4].

Breast cancer can be hereditary or sporadic. The most frequent mutations associated with hereditary cancer include those that affect DNA damage repair (DDR) genes, the most important of which are mutations in the BRCA1, BRCA2, and TP53 genes [5]. Sporadic cancer represents approximately 85% of all cases of breast cancer and is associated with some of the risk factors mentioned above; however, it has also been associated with exposure to carcinogens, such as air pollutants [6], electromagnetic radiation [7], and DDR gene expression dysregulation [8].

According to their presentation, ductal carcinoma *in situ* is the most diagnosed breast cancer type, followed by lobular carcinoma *in situ* [9]. Breast cancer, in turn, is divided into different subtypes based on the presence or absence of the estrogen receptor (ER), progesterone receptor (PR), and HER2 receptor. Thus, we can distinguish between a luminal subtype, being ER/PR+, an Her2+ subtype, which has this receptor overexpressed, and a triple negative or basal-like subtype (TNBC). Following this classification, the luminal subtypes can be divided into luminal A, characterized by ER/PR+, HER2–, and low Ki67 expression, and luminal B, characterized by ER/PR+, HER2+, and high Ki67 expression. Subtype Her2+ is ER/PR negative, and the triple negative indicates a lack of all these receptors [10–12].

Cell signal transduction is a fundamental process in the development and progression of cancer. Hanahan and Weinberg [13] noted that tumour cells exhibit a set of characteristics or hallmarks, including uncontrolled proliferation, genomic instability, and apoptosis evasion. To this end, modifications to various cell signalling pathways promote tumour cell proliferation, progression, and survival [14]. These alterations are due to mutations in oncogenes that overexpress certain proteins, mutated proteins that present uncontrolled activity, or inactivation of tumour suppressor genes that favour these processes [15].

**Figure 1:**
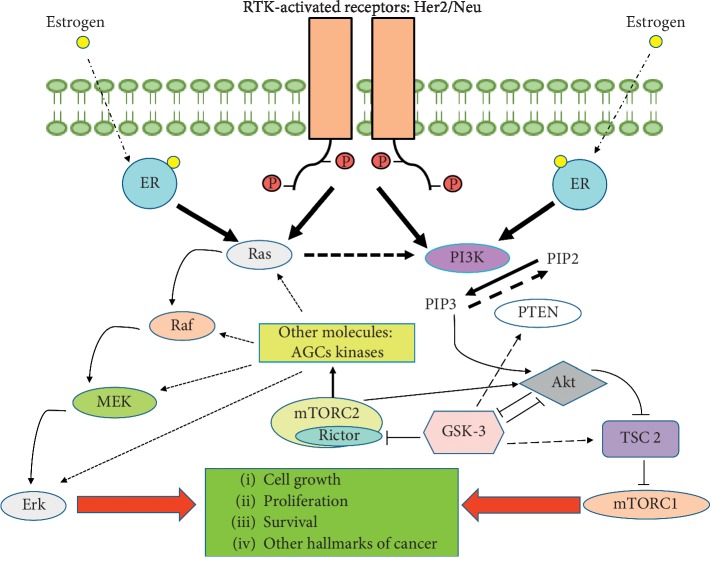
Overview of cell signalling mediated by the tyrosine kinase receptors (RTKs) Her2/Neu and estrogen receptors (ERs), two key components of breast cancer development. Their activation initiates the PI3K/Akt/mTOR and MAPK pathways, finally promoting cell growth, proliferation, survival, and other hallmarks of cancer. Although this is a review of PI3K/Akt/mTOR signalling, it is important to understand that the different pathways are connected by different points. In this figure, we have presented two examples: Ras, promoting PI3K activation, and how some AGC kinases (such as SGK-3) activated by mTORC2 also interact with the MAPK pathway. Additionally, GSK-3 plays an important role as well in the regulation of these pathways, represented in the figure. GSK-3 is an example of how complex those interactions are, by the inhibition and activation of different molecules implicated in PI3K and MAPK pathways.

Many alterations in breast cancer cells that affect cell signalling pathways have been described. In fact, variations have been described in the responses mediated by calcium-sensitive receptors [16, 17] or hypoxia-inducible factor [18] or even in the apoptotic cell mechanisms themselves [19]. However, the alterations most studied and most directly involved in the progression and development of breast cancer pathways are those mediated by the ER and human epidermal growth factor type-2 receptors (HER2/Neu or c-ErbB2) [19]. The activity of HER2 receptors in turn promotes the signalling of other pathways such as the mitogen-activated protein kinases (MAPKs) or cell components like glycogen synthase kinase-3 (GSK-3) and PI3K/Akt/mTOR pathways, both represented in Figure 1, denoting the importance of signal integration and transduction processes in the progression and development of breast cancer [20–23].

## 2. Importance of the PI3K/AKT/mTOR Pathway in Cancer

PI3K/Akt/mTOR is a cell signalling pathway involved in growth, proliferation, survival, motility, metabolism, and immune response regulation [24, 25]. This pathway has also been associated with a great variety of diseases and syndromes, such as tuberous sclerosis, Parkinson's disease, and vascular diseases [26–28].

Studies on PI3K/Akt/mTOR have also focused on cancer research. Alterations to this pathway have been found in practically all human tumours, including breast cancer, where up to 60% of the tumours present different variations that hyperactivate this pathway [29].

The dysregulation of this pathway has been related to a wide variety of cancer hallmarks, including uncontrolled proliferation, genomic instability, and metabolic reprogramming in tumour cells [13, 30]. In addition, PI3K/Akt/mTOR pathway activation is one of the main causes of cancer cell resistance to antitumour therapies [31]. This makes the PI3K/Akt/mTOR pathway a crucial object of study for understanding the development and progression of this disease, the role of this pathway as a potential therapeutic target, and the prognostic and diagnostic value of this pathway in patients with breast cancer [32, 33].

## 3. PI3Ks in Tumours and Existing Therapies

Phosphoinositide 3-kinases (PI3Ks) are a family of lipid kinases that integrate signals from growth factors, cytokines, and other extracellular stimuli and are related to the cell's response to these kinases [34]. Three different classes of PI3Ks are known, which in turn are divided into different subclasses according to their affinity for certain substrates, their sequence homology, and the functions that each of these classes have in cell signal transduction; class I PI3Ks are the most studied and the most clearly related to oncogenic processes [35]. Class I PI3Ks are divided into the following classes: PI3K IA, activated by receptor tyrosine kinases (RTK) receptors, G-protein-coupled receptors (GPCRs), and oncogenes such as Ras; and PI3K IB, regulated exclusively by GPCRs [36]. Among these, class IA is the most directly implicated in cancer. PI3K class IA consists of a regulatory subunit, which can be p85*α*, p55*α*, p50*α*, p85*β*, or p55*γ*, and a catalytic subunit, which can be p110*α*, p110*β*, or p110*δ* [37].

When a cellular receptor is activated, it dimerizes and autophosphorylates in various regions, phosphorylating, in turn, different adapter proteins and being recognized by the p85 subunit, which binds to these phosphorylated residues and generates a conformational change that releases p110, the subunit that exerts catalytic activity. Consequently, a phosphate group is added to phosphatidylinositol 4,5-bisphosphate (PIP2), transforming it into phosphatidylinositol (3,4,5)-trisphosphate (PIP3) [37]. Phosphatase and tensin homolog (PTEN) phosphatase, which can remove a phosphate from PIP3 to convert it into PIP2, is the most important negative regulator of this pathway and one of the tumour suppressors with the greatest effect on different types of cancer [38]. PIP3 levels can also be regulated by another tumour suppressor known as inositol polyphosphate-4-phosphatase type II B (INPP4B), which has the same effect as PTEN. INPP4B loss has been considered a marker of aggressiveness in TNBC tumours [39, 40].

**Figure 2:**
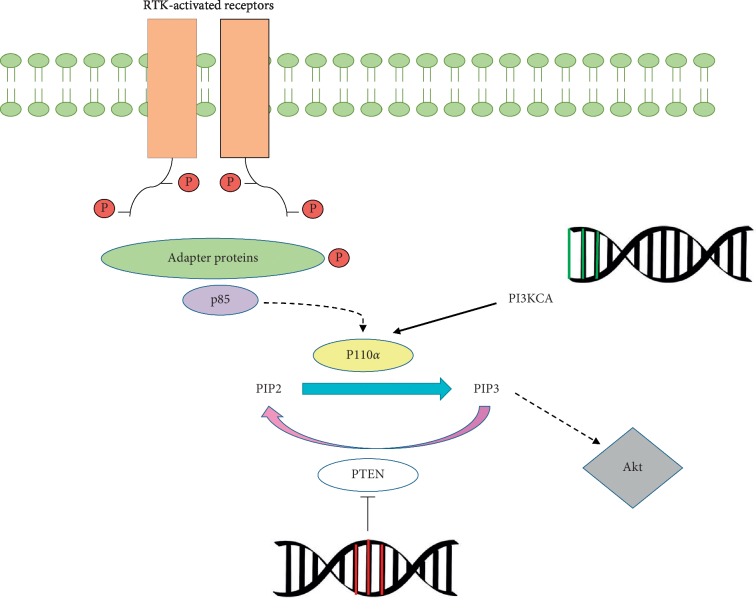
Mechanism of action of PI3K and PTEN. RTK receptors are activated, phosphorylating themselves and other series of adapter proteins, such as IRS-1. In this case, the regulatory subunit p85 binds to these residues and releases the catalytic subunit p110*α*, which adds a phosphate to PIP2 and transforms it into PIP3, which will subsequently activate Akt. PTEN prevents this activation by dephosphorylating PIP3. The loss of function of this gene, represented in red, or activating PI3KCA mutations, shown in green, can overactivate this route, favouring the development of cancer.

Activating mutations in PIK3CA, the gene that encodes the p110*α* catalytic subunit, have been identified as potent oncogenic mechanisms implicated in the hyperactivation of this pathway (Figure 2); these mutations are especially noteworthy in breast cancer, where up to 27% of patients have mutations in this gene [41]. Mutations in PI3KCA are more common in luminal A subtype cancers, where they are detected in 45% of tumours, followed by HER2+ mutations with a frequency of 39%; luminal B represents 30% of cancers, and TNBC alterations appear in 9% of cases [24]. These mutations affect mainly the helical domain of the p110α subunit, reducing its repression by regulatory subunits or facilitating its interaction with IRS1 [42, 43]. PI3K activation is a critical step in oncogenesis and plays a role in treatment resistance in ER+/HER2+ breast cancers. There are currently PI3K inhibitors, which are being implemented in clinical trials [44, 45].

Mutations in PTEN also result in important alterations in cell signalling in patients with breast cancer. The relationship between the loss of PTEN gene function and unfavourable predictive factors has been observed in various types of cancer, such as gastric, prostate, and colorectal cancer [46–48]. Lu et al. [49] described how the activity exerted by this phosphatase slows cell growth and induces apoptosis and anoikis in breast cancer cells. The results of a meta-analysis of 27 studies and 10,231 cases performed by Li et al. [50] showed that there seems to be an association between PTEN expression loss and tumour aggressiveness in breast cancer patients, especially those with ER, PR, and TNBC tumours, thus demonstrating the involvement of PTEN in the initiation and malignancy of breast cancer tumour cells.

There are multiple PI3K inhibitors that have been developed or are in the study phase. These are classified according to their specificity for each isoform and can be divided into (1) first-generation inhibitors, also known as pan-inhibitors of PI3K, which target all the different PI3K class I isoforms (*α*, *β*, *δ*, or *γ*), (2) second-generation inhibitors, which are specific to any of these isoforms, or (3) dual PI3K/mTOR inhibitors. However, the efficacy shown by these types of inhibitors as therapeutic agents is far from that expected due to the coexistence of various mutations present in tumour cells, compensatory feedback cycles, or the toxicity associated with these treatments [24]. That is why research has focused on these inhibitors in combination with other drugs, although much work remains.

Of the first-generation inhibitors, breast cancer studies have focused on the use of pictilisib and buparlisib. The role of pictilisib as a pan-inhibitor (GDC-0941) in the inhibition of the metastatic phenotype in thyroid carcinomas has been demonstrated due to its action on PI3K and HIF-1*α* [51]. Zou et al. [52] observed the synergistic role that this drug had with the MEK inhibitor UO126 in inhibiting NSCLC cell growth. A study conducted by Schmid et al. [53] showed how the combination of pictilisib with anastrozole obtained a better response in inhibiting breast cancer cell proliferation. However, in a study conducted by Krop et al. [54], the combined use of pictilisib with fulvestrant was not associated with an improvement in the treatment of patients with advanced-stage breast cancer resistant to endocrine therapy, regardless of the presence or absence of PI3KCA mutations, measured in terms of progression-free survival (PFS).

Buparlisib (BKM120), another pan-inhibitor of PI3K, has demonstrated synergistic growth inhibitory effects in combination with agents targeting the HER2 receptor in preclinical studies [45]. Several early clinical trials have shown positive results for the use of buparlisib in breast cancer patients [55–57]. Another phase II clinical trial developed by Loibl et al. [58] studied the combination of buparlisib with trastuzumab and paclitaxel in women with primary HER2+ tumours. The results showed little reliability in its use; however, high rates of objective response and a reduction in the Ki67 cell proliferation marker in the ER+HER2+ subgroup denote the importance of future investigations of therapies targeting this pathway. Furthermore, two important clinical trials need to be mentioned here: BELLE-2 and BELLE-3. Both were double-blind, randomized, placebo-controlled phase 3 trials in which the use of buparlisib was analysed in hormone receptor-positive Her2-negative postmenopausal women. BELLE-2 aimed to evaluate the combination of buparlisib plus fulvestrant versus placebo and fulvestrant. The results showed that the use of this PI3K inhibitor combined with endocrine therapy was associated with a significant improvement compared to fulvestrant alone. However, this combination was also found to have considerable toxicity, limiting its efficacy [59]. In BELLE-3, 432 postmenopausal women who had previously received endocrine therapy and mTOR inhibitors were divided again into two groups: a group that received fulvestrant and buparlisib and a control group that received only fulvestrant. Patients with fulvestrant plus buparlisib had a longer PFS than those who received fulvestrant alone, but again, the toxicity of this combination restricts its use [60]. On the whole, these works highlight the importance of performing further studies with PI3K-*α*-specific inhibitors to provide the greatest benefits.

Currently, research is also aimed at the use of second-generation PI3K inhibitors, such as alpelisib (BYL719) and taselisib (GDC0032), which target the PI3K-*α* isoform, as a higher-safety profile is expected compared to previous inhibitors [61]. Numerous previous studies seem to indicate that mutations in PI3KCA in ER + tumours decrease the response rate of breast cancer tumours to antiestrogenic therapy [62, 63]. A series of preclinical cell models developed by Fritsch et al. [64] demonstrated how cells with mutations in PIK3CA had greater sensitivity to alpelisib, while cell lines without this mutation had selective sensitivity. A phase III clinical trial showed how patients with advanced tumours and mutations in PIK3CA benefited from the use of alpelisib in combination with fulvestrant; compared with fulvestrant with placebo, this combination increased the PFS and response rates [65]. In fact, alpelisib has recently become the first PI3K inhibitor approved by the FDA for the treatment of breast cancer, so the study of this type of inhibitor is a growing point of interest in actual research. Other preclinical studies also reported the efficacy of taselisib in cellular models with mutations in PIK3CA in breast cancer [66]. SANDPIPER is an ongoing randomized, double-blind phase III clinical trial with 631 postmenopausal women with ER + HER2- PI3KCA mutations who showed progression or recurrence during or after aromatase inhibitor (AI) therapy. This trial aims to investigate the efficacy and safety of taselisib plus fulvestrant in comparison to a fulvestrant alone control group (NCT02340221). SOLAR-1 is another ongoing phase III clinical study designed to evaluate the use of alpelisib plus fulvestrant in ER/PR + Her2– advanced cancer patients after treatment with AI (NCT02437318). Currently, GDC-0077, another second-generation PI3K-*α* inhibitor, is being tested in a phase I clinical trial in patients with PIK3CA mutant solid tumours that are locally advanced or metastatic, including breast cancer, and GDC-0077 is being tested in combination with targeted therapies focused on the ER/PR+Her2– subtype (NCT03006172).

Regarding third-generation inhibitors or dual PI3K/mTOR inhibitors, the role of some of these inhibitors, such as BEZ235, has been studied in combination with other inhibitors, such as everolimus (RAD-001), and these inhibitors have been shown to exert a synergistic action by decreasing the growth of some TNBC cell lines, such as MDA-MB-231 cells or the ER + MCF-7 line [67]. The combination of BEZ-235 with autophagy inhibitors has also been studied, and these combinations have resulted in inhibited proliferation and increased apoptosis in breast cancer cells [68]. However, clinical studies of BEZ-235 have not yielded promising outcomes. A phase I/IB study of BEZ-235 in patients with Her2+ advanced breast cancer showed that the safety profile of this inhibitor was not adequate [69]. Nevertheless, clinical studies of BEZ235 are ongoing in patients with metastatic breast cancer [70]. Some previous studies indicate how AIs can be combined with PI3K/mTOR inhibitors for treating patients with resistance to endocrine therapy who present with ER+HER-metastatic cells. In addition, it has been studied how the PF-04691502 inhibitor is currently used for combined treatment with tamoxifen for treating breast cancer stem cells (BCSCs); in this manner, the resistance they presented when tamoxifen was applied in the absence of another drug was avoided, and the importance of this pathway in therapy resistance and in the formation of mammals was shown [71]. Finally, another dual PI3K/mTOR inhibitor, GDC-0980, has been shown to be particularly effective in BRCA-competent TNBC when combined with a PARP inhibitor and carboplatin because it inhibits the DDR system [72]. Studies on the efficacy of DDR inhibitors in breast cancer therapy are discussed in previous reviews [73].

## 4. AKT in Tumours and Existing Therapies

RAC-alpha serine/threonine-protein kinase (Akt), also known as protein kinase B (PKB), is a serine-threonine kinase that has three different isoforms encoded by three different genes: Akt1, Akt2, and Akt3. However, it is Akt1 that has been most associated with cancer [74]. Akt1 binds to PIP3 via pleckstrin homology (PH) domains [75]. This interaction localizes Akt to the cell membrane, resulting in the subsequent phosphorylation of the Thr308 and Ser473 residues by 3-phosphoinositide-dependent protein kinase-1 and -2 (PDK1 and PDK2). While PDK1 is activated by PIP3, PDK2 forms part of the mTORC2 complex [76].

**Figure 3:**
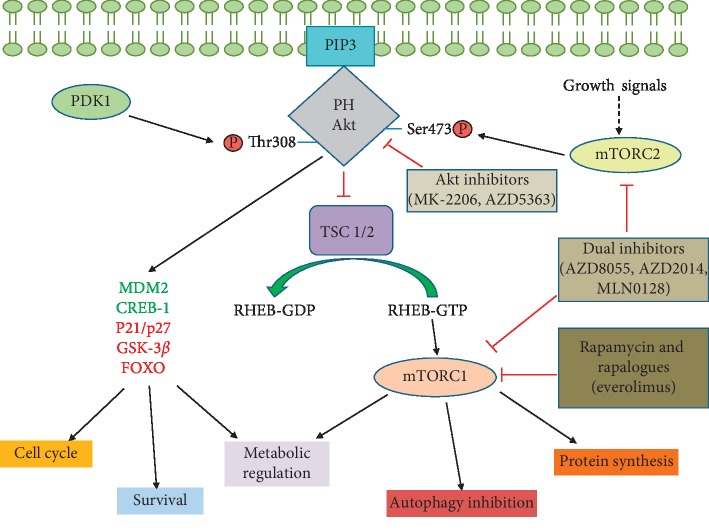
Activation of Akt after joining to PIP3 by its PH domain. PDK1 and PDK2, present in mTORC2, phosphorylate Akt, which will activate and inhibit a series of genes, transcription factors, and proteins, represented in red, such as the TSC complex, which will eventually activate mTORC1, resulting in a series of cellular responses. We have remarked in green the components which are stimulated or overactive in these cells. This figure also shows the main therapies developed to focus on this pathway.

The effects of Akt activation include the activation of MDM2, a p53 inhibitor, and the inhibition of other proteins involved in stimulating cell survival in stress situations [77]. The activation of Akt has also been associated with the inhibition of p21 and p27, which are involved directly in cell cycle control [78], and GSK-3*β*, as shown in Figure 3. GSK-3*β* inactivation is directly related to cell metabolism reprogramming and, more specifically, to the uptake and use of glucose, which favours the Warburg effect [79]. However, the role of GSK-3*β* in cancer remains controversial. Some studies have reported that GSK-3*β* may function as a tumour promoter. GSK-3*β* knockdown cells were associated with decreased levels of Bcl-2 and VEGF, thus suggesting the role of GSK-3*β* in inhibiting apoptosis and promoting angiogenesis [80]. On the other hand, previous findings have indicated GSK-3*β* as a tumour suppressor in breast cancer because it increases the sensitivity of chemotherapy and inhibits PI3K/Akt and Wnt signalling, thus playing a key role in the cell cycle and survival [81, 82]. The interaction of GSK-3 with PI3K/Akt pathway is represented in Figure 1. The activation of Akt is also related to the activation of mTORC1. In addition, Akt is involved in the activation and inactivation of various transcription factors; for example, it binds 14-3-3 proteins and prevents the translocation of FOXO to the nucleus [83]. Akt is also important in the regulation of NF-*κβ*-dependent gene transcription and CREB-1 overactivation, thus promoting the expression of antiapoptotic genes such as Bcl-2 and mcl-1 [84, 85].

The hyperactivation of Akt and consequently mTOR may facilitate the resistance that some patients with breast cancer have to endocrine therapies; in these patients, an inverse correlation was established between Akt activation and a partial treatment response [86, 87].

In breast cancer, the most frequent mutation is E17K-Akt1, representing 2% of all breast cancers. This mutation increases the affinity of the PH domain for lipids, resulting in the constitutive localization of Akt in the cell membrane [88]. Currently, there are phase I and II clinical trials with allosteric and catalytic Akt inhibitors. Hyman et al. [89] proposed the use of the E17K-Akt1 mutation as a predictive marker of the response to the catalytic inhibitor AZD5363, with promising results. However, studies with the allosteric inhibitor MK-2206 combined with hormone therapy reported benefit ratios of only 42% [57].

In addition, recent studies indicate that there are other proteins also activated by PI3K that contribute to the development of cancer. Lien et al. [30] described that Akt is not always hyperactivated in the presence of PI3KCA or PTEN mutations; the authors also showed the role that other PI3K-dependent proteins may play in the resistance to pathway inhibitors and how these proteins can substitute for Akt-mediated signalling, indicating that further knowledge regarding these pathways is important for developing promising future therapies (Figure 3).

## 5. mTOR in Tumours and Existing Therapies

The mammalian target of rapamycin (mTOR) is a protein kinase comprising two distinct complexes, mTORC1 and mTORC2. Both complexes are associated with a set of proteins that can be common or specific to each complex [90]. mTORC1 is a complex formed by the mTOR, Raptor, Deptor, Pras40, and mLST8 proteins [91]. This complex is activated by Akt via inhibition of the tuberous sclerosis complex (TSC) [92]. In turn, TSC inhibits mTORC1 due to its ability to inactivate RHEB, a GTPase that activates this complex. mTORC1 responds to amino acids, stress, oxygen levels, energy needs, and growth signals [24]. The activation of mTORC1 seems to be involved in multiple cellular processes, including protein synthesis control, through phosphorylation of the ribosomal proteins S6K1 and 4E-BP1, the regulation of metabolism, and the inhibition of autophagy [93].

mTORC2 is a complex that shares certain components with mTORC1, such as mTOR kinase, mLST8, and Deptor. In addition, it contains the Rictor protein and the subunits Sin1 and Protor 1/2 [91]. This complex is insensitive to rapamycin, unlike the mTORC1 complex [94]. mTORC2 is activated by growth signals and controls cell proliferation and survival processes, as well as the cytoskeleton, mainly by phosphorylating other proteins such as Akt, which, in turn, is the main activator of mTORC1 [95]. In fact, mTORC2 phosphorylates AGC kinase family members, which are Akt, SGK, and PKC (Figure 1), all of which have oncogenic effects [96, 97]. The role of SGKs is also interesting in cancer because they interact at various levels with MAPK signalling and are related to the tumourigenesis process in PI3K mutants through Akt-independent but SGK-3-dependent malignant transformation (Figure 1) [98]. mTORC2 also interacts with the retinoblastoma (Rb) tumour suppressor. Previous studies have demonstrated that Rb may inhibit Akt activation by inhibiting the mTORC2/PDK1 phosphorylation of Ser-473 Akt [99]. Furthermore, mTORC2 has been associated with the control of the expression of glycolytic genes by epigenetic regulation of H3K56Ac levels in glioma cells [100], suggesting the different and important roles that this complex may have in cancer progression and regulation.

Everolimus is one of the main drugs that targets mTOR, and numerous studies have demonstrated its effectiveness in breast cancer therapy [101]. Du et al. [102] reported the efficacy of everolimus in the inhibition of antiapoptotic proteins such as Bcl-2, as well as in breast cancer cell cycle and growth arrest and in disease progression. Other studies have shown the efficacy of everolimus in the treatment of advanced ER + PR + breast cancer [103]. Everolimus is an allosteric inhibitor of mTORC1 but not mTORC2 and can increase Akt phosphorylation by not binding the latter inhibited complex. Dual inhibitors of mTOR, affecting both complexes, have been studied. Leung et al. [104] demonstrated *in vitro* the synergy that this drug presented with other pathway inhibitors, such as dual mTOR inhibitors, representing new strides for greater efficacy of the developed therapies targeting this pathway.

Among the dual inhibitors are AZD8055, AZD2014, and MLN0128, which are in clinical development but have already demonstrated efficacy in numerous studies [105, 106]. These inhibitors show better results compared to mTORC1 inhibitors in blocking the PI3K/Akt/mTOR pathway as measured by 4-EBP1, SK6, and p-Akt inhibition [107]. The effectiveness of these inhibitors has been demonstrated even in everolimus resistance acquired by mutations in the rapamycin binding domain of mTOR (Figure 3) [108]. Here, it is important to discuss the role of mTOR inhibitors and their relationship with the DNA-dependent protein kinase catalytic subunit (DNA-PKcs), another key component of the DDR system. Low levels of DNA-PKcs protein expression were related to higher tumour grade, dedifferentiation and mitotic index, and poor survival [73, 109]. CC-115, a dual inhibitor of mTOR and DNA-PKcs, has been shown to inhibit cell growth *in vitro* by blocking DDR pathways, thus inducing apoptosis in many cancer lines, including breast cancer cells [110]. Currently, a phase I clinical trial with CC-115 is ongoing. Other studies also report the role of the DNA-PK inhibitor NU7441 in sensitizing breast cancer cells to ionizing radiation and doxorubicin [111].

Recently, an mTORC2-selective inhibitor based on nanotechnology for HER2 amplification combined with the HER2 inhibitor lapatinib was validated in TNBC, showing its promising efficacy for both subtypes [112].

## 6. IRS4 in Tumours and the Relationship with PI3K/Akt/mTOR in Breast Cancer

Insulin receptor substrates (IRSs) are a set of adaptive cytoplasmic proteins that were originally identified by their role in insulin signalling [113]. The IRS family comprises six members. IRS1 and IRS2 are the best studied variants due to their broad expression in different tissues, and they strongly resemble each other in their structures [114]. IRS3 has been found in rodents but not in humans [115]. IRS5/DOK4 and IRS6/DOK5 are two distant members of the family with greater resemblance to each other than to the other IRSs [116]. The present study will review IRS4, which has been implicated in some types of cancer, such as breast cancer, squamous carcinoma of the lung, sarcomas, and acute lymphoblastic leukaemia [117, 118].

IRS phosphorylation occurs at tyrosine residues, generally through RTK-type receptors, thus inducing a signalling cascade that, in turn, promotes the activation of other pathways such as PI3K/Akt or MAPK (Figure 1) [114, 119]. However, Ikink et al. [120] demonstrated that IRS4, unlike other family members, did not require growth factors, leading to the proliferation of mammary epithelial cells due to elevated basal activation of the PI3K/Akt/mTOR pathway. This activation is achieved through the interaction of IRS4 with the p85 subunit [121]. In addition, an association was found between adenovirus infection or certain retroviruses and increased levels of IRS4 [121, 122].

Other studies have shown that there is a positive correlation between IRS4 levels and other proteins involved in breast cancer, such as Breast Tumor Kinase (Brk), a kinase that is overexpressed in 80% of breast tumour cells, indicating the importance of this protein in the development of breast cancer [123, 124].

It has also been observed that IRS4 induces resistance to trastuzumab and lapatinib, two treatments directed against HER2+ breast cancer cells, because it is involved directly in the hyperactivation of the PI3K/Akt/mTOR pathway, which is ultimately responsible for this resistance, as shown in previous studies; thus, IRS4 is a promising therapeutic target for these types of tumours that are resistant to treatment [125, 126].

## 7. Future Directions

The study of cellular events that occur in oncogenic processes is essential to understand the treatment actions we can take. Furthermore, it is important to understand that carcinogenesis involves a wide range of changes in tumour cells that enable their malignant transformation. From this perspective, the PI3K/Akt/mTOR cell signalling pathway is one of the key points of study that could result in improvements in the existing survival curves after good anatomopathological studies and analyses of the efficacy and efficiency of the inhibition. Despite the existence of selective PI3K/Akt/mTOR inhibitors and current clinical trials, the underlying cellular mechanisms are not yet known. Studying the existing scientific literature helps to understand the current state and the possible paths to be taken. To maximize the efficacy of the inhibitors and decrease their toxicity, PI3K/Akt/mTOR targeting is commonly combined with additional treatments, such as endocrine therapy or DDR inhibitors. Current challenges may be to minimize the side effects of these therapies as well as to increase their specificity, thus bringing more benefits to the patients who need to receive this type of treatment.
